# Changes in clinical and radiographic parameters after a regimen of chiropractic manipulation combined with soft tissue therapy and neuromuscular rehabilitation in 7 patients with adolescent idiopathic scoliosis

**DOI:** 10.1186/1748-7161-8-S1-P5

**Published:** 2013-06-03

**Authors:** AJ Woggon, DA Martinez

**Affiliations:** 1 CLEAR Scoliosis Institute of Texas, Dallas, TX, USA; 2 Independent Researcher, Dallas, TX, USA

## Background

The causes of idiopathic scoliosis (IS) are likely multifactorial, including genetic and environmental.[1] It is unlikely one therapy addresses all involved factors. Evidence supports a comprehensive approach to evaluation & treatment using a variety of outcome assessments.[2]

## Aim

This study presents a review of files of seven adolescent idiopathic scoliosis (AIS) patients treated with a comprehensive two-week treatment protocol including chiropractic manipulative therapy, massage, exercise, and whole-body vibration therapy, followed by a home rehabilitation regimen.

## Method

Primary outcome measures reported include Cobb angle, disc index, apical vertebral deviation, vertebral rotation, digital spirometry, scoliometry, timed one-legged stability with eyes closed (TOLSWEC), and computerized dual inclinometry, as well as pain drawings and health-related quality of life questionnaires (RAND SF-36 and SRS-22). Data was recorded post-treatment and at follow-up ranging from four to seven months. A paired t-test and Wilcoxon test was performed to assess the statistical significance of the pre and post treatment radiographic parameters. Each patient underwent twenty treatment sessions over a two week period (2x day/five days) for an average length of 180 minutes/session. Treatment sessions were divided into three phases. The first phase of treatment addressed soft tissue deformations and improving spinal flexibility, the second phase influenced spinal biomechanics, and the third impacted neuromuscular function.

## Results

The following changes were noted post-treatment: Cobb angle, 0 to 12 degrees; disc index, -0.11 to 0.44; apical vertebral deviation, -21.5 mm to 13 mm; vertebral rotation, 0% to 19.6%; forced vital capacity, 0 cc’s to 820 cc’s; forced expiratory volume in 1 second, -50 cc’s to 520 cc’s; peak expiratory flow, -960 cc’s to 1180 cc’s; forced expiratory rate, -2% to 18%; scoliometry, -1 to 10 degrees; TOLSWEC, -8 to 24 seconds; spinal ranges of motion, -4.5 to 29 degrees; pain scale, +1 to -4; and, RAND SF-36 scores, -21% to 36%. The median changes pre and post –treatment were significant (p<0.05) for Cobb angle, apical vertebral rotation, thoracic Disc Index, and lumbar apical vertebral deviation. Three patients maintained some degree of improvement at follow-up, and two demonstrated continued improvement. One patient was lost to follow-up.

## Conclusion

The applied protocols effected positive functional and/or radiological changes in seven cases of AIS, with two cases demonstrating continued benefit at follow-up. Additional research is needed to determine the benefit of this multifactorial approach.
